# Muscle weakness is associated with non-contractile muscle tissue of the vastus medialis muscle in knee osteoarthritis

**DOI:** 10.1186/s12891-022-05025-1

**Published:** 2022-01-27

**Authors:** Josien C. van den Noort, Marike van der Leeden, Gerard Stapper, Wolfgang Wirth, Mario Maas, Leo D. Roorda, Willem F. Lems, Joost Dekker, Martin van der Esch

**Affiliations:** 1 Department of Radiology and Nuclear Medicine, Amsterdam Movement Sciences, Medical Imaging Quantification Center (MIQC), Amsterdam UMC, Univ of Amsterdam, Meibergdreef 9, Amsterdam, Netherlands; 2 Department of Rehabilitation Medicine, Amsterdam Movement Sciences, Amsterdam UMC, Vrije Universiteit Amsterdam, de Boelelaan 1117, Amsterdam, Netherlands; 3 Amsterdam Rehabilitation Research Center Reade, Amsterdam, Netherlands; 4 Department of Rehabilitation Medicine, EMGO Institute for Health and Care Research, Amsterdam UMC, Vrije Universiteit Amsterdam, de Boelelaan 1117, Amsterdam, Netherlands; 5 Institute of Anatomy, PMU, Salzburg, Austria; 6 Chondrometrics GmbH, Ainring, Germany; 7 Department of Rheumatology, Amsterdam UMC, Vrije Universiteit Amsterdam, de Boelelaan 1117, Amsterdam, Netherlands; 8 Department of Rheumatology, Amsterdam Rehabilitation Center Reade, Amsterdam, Netherlands; 9 Department of Psychiatry, Amsterdam UMC, Vrije Universiteit Amsterdam, de Boelelaan 1117, Amsterdam, Netherlands; 10 Amsterdam University of Applied Sciences, Faculty of Health, Amsterdam, Netherlands

**Keywords:** Muscle weakness, Knee osteoarthritis, MRI, Muscle, Vastus medialis

## Abstract

**Background:**

Quadriceps weakness is assumed to be associated with compositional properties of the vastus medialis muscle in patients with knee osteoarthritis (OA).

**Methods:**

The aim was to determine the association of non-contractile muscle tissue in the vastus medialis muscle, measured with routine MRI, with muscle extensor strength in patients with knee OA. Sagittal T1-weighted 3T MRI of 94 patients with knee OA, routinely acquired in clinical practice were used for analysis. Using the MRI’s, the amount of non-contractile muscle tissue in the vastus medialis muscle was measured, expressed as a percentage of (non)-contractile tissue, dichotomized into a low and a high non-contractile percentage group. Muscle strength was assessed by isokinetic measurement of knee extensors and by conduction of the Get-Up and Go (GUG) test. In regression analyses, associations of percentage of non-contractile muscle tissue with muscle strength and GUG time were determined and controlled for sex, age, BMI and radiographic severity.

**Results:**

A high percentage of non-contractile muscle tissue (> 11.2%) was associated with lower muscle strength (B = -0.25, *P* = 0.006) and with longer GUG time (B = 1.09, *P* = 0.021). These associations were specifically confounded by sex and BMI, because these two variables decreased the regression coefficient (B) with > 10%.

**Conclusions:**

A high percentage of non-contractile muscle tissue in the vastus medialis muscle measured by clinical T1-weighted 3T MRI is associated with muscle weakness. The association is confounded by sex and BMI. Non-contractile muscle tissue seems to be an important compositional property of the vastus medialis muscle underlying quadriceps weakness.

## Introduction

Muscle weakness is often present in patients with knee osteoarthritis (OA) [1–3]. Improvement in muscle strength is a corner stone in treatment guidelines of knee OA [4]. However, effects of improved muscle strength on pain, physical function and quality of life in knee OA are, although significant, moderate [1, 5–8]. A better understanding of the underlying mechanisms of muscle weakness can help to improve muscle strength training. Possible explanation for muscle weakness is a reduction in muscle quantity: muscle mass and cross sectional area [9–11]. Muscle weakness, however, can also be reflected in muscular compositional properties affecting muscle strength [12–14]. Several studies investigated the association between muscle weakness and muscle composition: in knee OA [14], healthy subjects [15, 16] and elderly [17–19].

The vastus medialis is a functionally important part of the quadriceps which supports the knee extension [12, 20]. Like all muscles, the vastus medialis is composed of two tissue components: 1) contractile tissue consisting of muscle cells and 2) non-contractile tissue which consists of fatty tissue and connective tissue [21]. A higher percentage of non-contractile muscle tissue in the vastus medialis is related to a higher risk for knee OA progression [12, 20]. In knee OA, intramuscular fat in the quadriceps is higher compared to controls and related to disease severity [14, 22]. Furthermore, the intramuscular fat volume of the thigh muscles contributes to changes in strength of the knee extensor and physical performance in women with knee OA [13]. Therefore, it is to be expected that a high percentage of non-contractile tissue in the vastus medialis is related to extension weakness and poor physical function.

Magnetic resonance imaging (MRI) is particularly useful to image soft tissues such as muscles and adipose tissue. For knee OA, MRI is currently acknowledged as the most important imaging modality in research, and increasingly advocated to be used in clinical trials and clinical practice [23, 24]. MRI with a chemical shift-based water/fat separation method has been used [14–16] to investigate the association between muscle weakness and a high percentage of non-contractile tissue in knee OA and healthy subjects. This MRI method is currently one of the most advanced and accurate techniques for fat imaging [25]. However, such advanced MRI methods are not routinely used in clinical practice for knee OA. An MRI method clinically used for imaging the knee joint is T1-weighted 3 Tesla (3T) MRI [26]. It is used for clinical purposes to examine the degeneration of the knee joint and not primarily to analyze muscle tissue. Separation of the various components of non-contractile tissue, i.e. intra-muscular fatty, connective and inflammatory tissue is not possible. However, these clinical MRI’s are easily available and might be useful to measure the percentage of the total amount of non-contractile tissue in the visible muscle tissue [11, 20, 21].

Therefore, the aim of this study was to determine the association of non-contractile muscle tissue in the vastus medialis muscle, measured by clinically available T1-weighted 3T MRI, with muscle strength represented by isokinetic muscle extensor strength and the Get-Up and Go test, in patients with clinically diagnosed knee OA.

## Methods

### Participants

Participants were recruited from a randomized controlled knee joint stabilization trial, involving 159 knee OA patients [27]. Participants were referred to a tertiary rehabilitation center with knee and/or hip complaints due to OA [27]. Of the 159 participants, MRI’s of the knee joint of 94 participants were available for this study. The MRI’s were taken at baseline, prior to the trial. From the included participants, the index knee was imaged, i.e. the knee that most severely affected daily activities based on pain and showed the highest radiographic severity. Inclusion criteria were clinical knee OA diagnosis according to the American College of Rheumatology criteria (knee pain, morning stiffness < 30 min duration, crepitus on active motion, tenderness of the bony margins of the joint, bony enlargement noted on examination, and a lack of palpable warmth of the synovium [28]), age between 40 and 75 years, and written informed consent [27]. Exclusion criteria were total knee arthroplasty, any form of arthritis other than OA, comorbidities that affect daily functioning, severe knee pain (numeric rating scale (NRS) > 8) and contra-indication for MRI (e.g., pacemaker, claustrophobia). Participants were examined by radiologists, rheumatologists and physiatrists. The study was approved by the local Institutional Review Board (29 September 2008, U/2782/0851). The protocol was performed in accordance with the relevant guidelines and regulations.

### Measurements

#### Muscle strength

Knee extensor torque, used as a measure of muscle strength, was assessed by using an isokinetic dynamometer (EnKnee, Enraf–Nonius, Rotterdam, Netherlands), at 60 ^o^/sec between 20–80° of knee extension, using a standardized protocol as described in Van der Esch et al. [29]. Patients were seated on a bench and secured to the testing device using straps to limit contributions from other muscle groups. The mechanical axis of the dynamometer was aligned with the knee joint. After a warming up period (in which a few non maximal contractions were practiced), participants performed three concentric knee extensions of the index knee at maximal strength with a rest period of about a minute between the repetitions. Verbal motivation during the repetitions was kept constant among all participants. Visual feedback was not provided. The mean of the maximum muscle strength of three valid repetitions was calculated, normalized to body weight, and used for further analyses (in Nm/kg). Repetitions were excluded when not correctly performed or when patients experienced pain.

The Get-Up and Go test (GUG), a physical-performance based test, was used as a surrogate measure of knee muscle strength [30]. The time it took for the participant to stand up from a standard-height chair and walk 15 m as fast as possible was measured (in seconds). Participants could wear their own shoes and use a cane if required for walking. A longer time to complete the GUG test represents increased functional disability/limitations [29] and in the present study muscle weakness.

#### Magnetic resonance imaging (MRI)

MRI scans of the index knee were clinically available and performed by a 3 Tesla whole-body scanner (General Electric Medical Systems, Milwaukee, WI, USA) using a phased array knee coil [31]. A sagittal T1-weighted turbo spin-echo was used. Scan parameters were: field of view of 180 mm, slice thickness 3 mm; interslice gap 0.3 mm; repetition time 760 ms; echo time 14 ms; turbo factor 2; matrix 384 × 256. The MRI scans were acquired in clinical practice with the aim to observe the knee joint and soft tissues around it to examine the degeneration of the knee joint [23, 24]. It also provided the possibility to study the muscle tissue. The middle slice of all slices in which the vastus medialis muscle was partly visible, was used for analysis.

#### Region of Interest

**Fig. 1:**
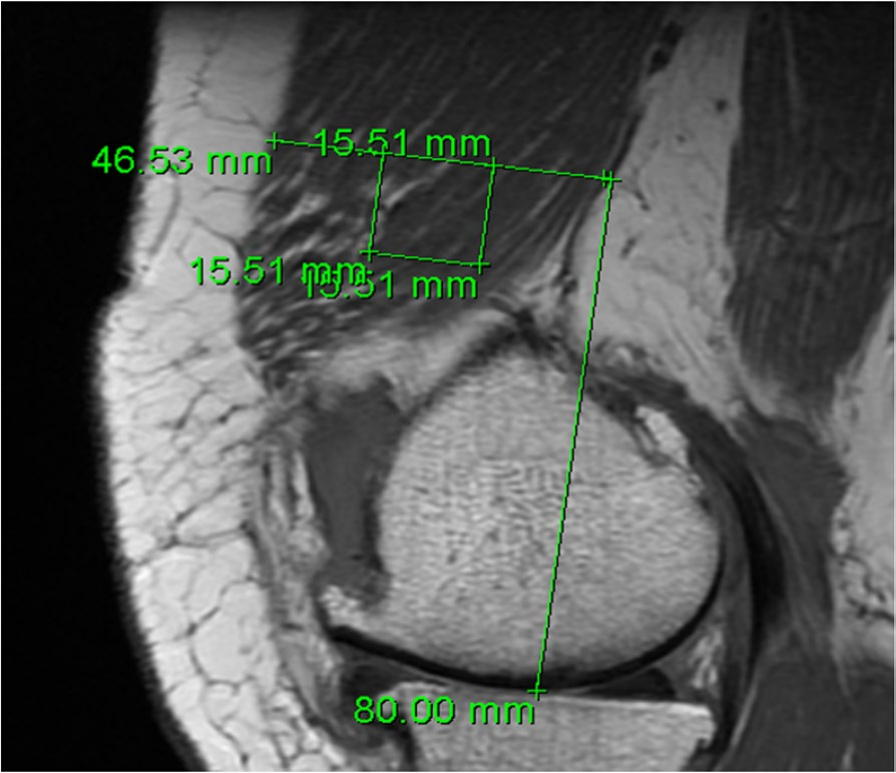
Location of the region of interest (ROI) in the vastus medialis muscle in a sagittal T1-weighted magnetic resonance image (3 T). The ROI was set 8 cm above the joint space, in the middle of the vastus medialis muscle

To quantify non-contractile tissue in the vastus medialis muscle on the MRI, a region of interest (ROI) was used. Centricity® Dicom Viewer 3.1 was used to set this ROI in the selected image slice. First, the thickness of the vastus medialis muscle was measured at 8 cm above the joint space, orthogonal to the long axis of the femur [20]. The standardized location and size of the ROI was dependent on the thickness of the vastus medialis muscle. Second, the size of each of the sides of a square ROI was calculated as one-third of the thickness of the measured muscle. Finally, the precise location of the ROI was set under two conditions: 1) the top edge of the ROI should be located at 8 cm above the joint space, orthogonal to the long axis of the femur and 2) the ROI should be located in the middle of the vastus medialis muscle on a sagittal axis, leaving a third of the muscle thickness on both sides of the ROI (Fig. 1). This standardized ROI was used for analysis. Reproducibility of placement of the ROI was assessed by two raters in 12 scans, showing intraclass correlation coefficients of 0.89 and 0.90 for the vastus medialis thickness and the percentage of non-contractile tissue, respectively.

#### Non-contractile tissue quantification

**Fig. 2:**
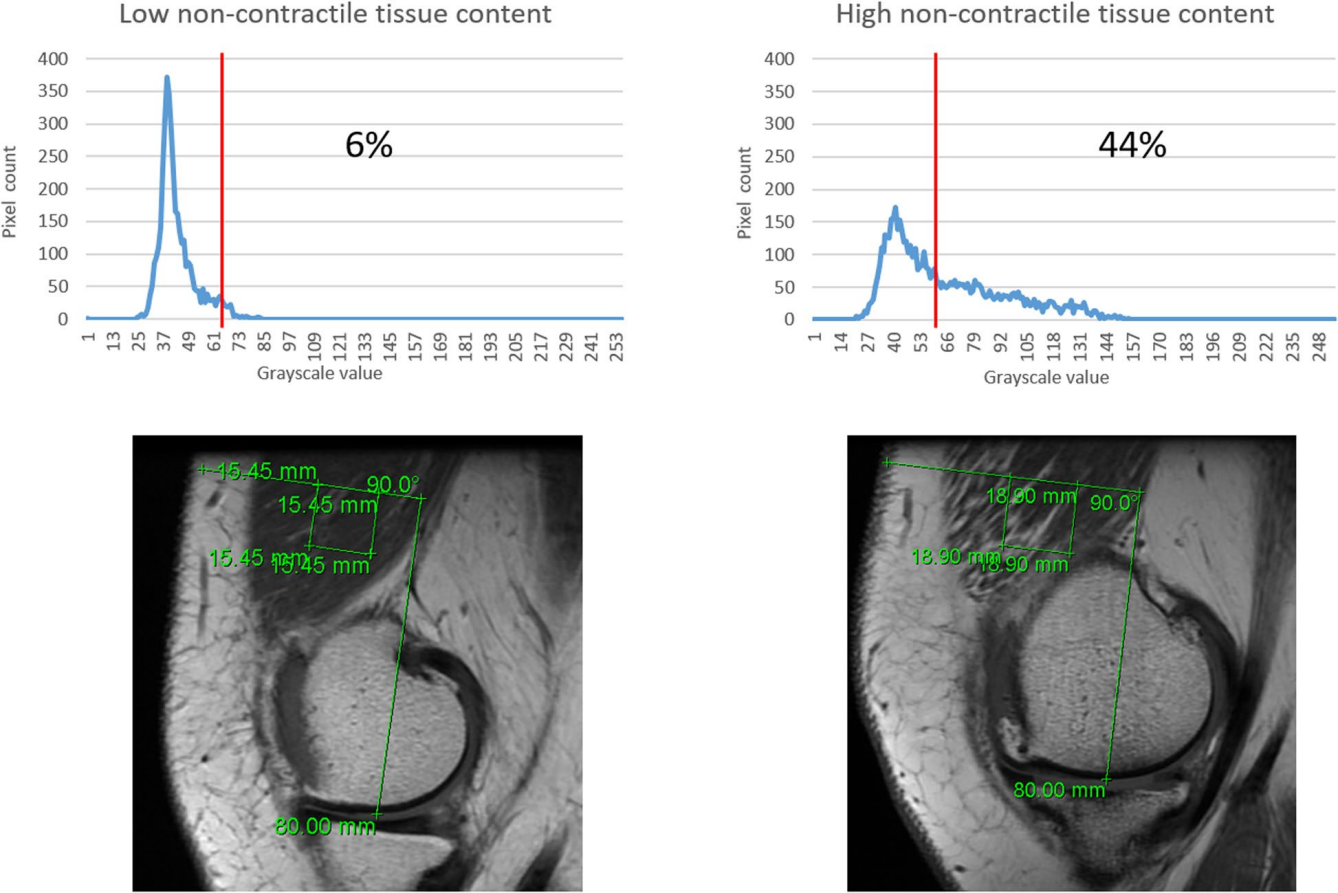
Quantification of non-contractile tissue was performed by analyzing grayscale values of pixels within the region of interest (ROI). The graphs represent examples of the distribution of pixels based on grayscale values in the ROI from the corresponding MRI’s (low and high non-contractile tissue content). A threshold was set at a grayscale value of 60. Grayscale values ≤ 60 were considered to present contractile tissue and grayscale values > 60 were considered to present non-contractile tissue. The percentage of non-contractile tissue was calculated as the number of non-contractile pixels (grayscale value > 60) divided by the total number of pixels

Quantification of non-contractile tissue was performed by analyzing grayscale values of pixels within the ROI using the program ImageJ (http://imagej.nih.gov/ij/, US National Institutes of Health, Bethesda, Maryland, USA). From Dicom Viewer, the images including the ROI were exported to ImageJ. A graph represented the distribution of pixels within the ROI based on grayscale values (Fig. 2): light grayscale values are non-contractile tissue and dark grayscale values are contractile muscle tissue (range from 0–255). A threshold was set at a grayscale value of 60, with ≤ 60 representing contractile tissue and > 60 representing non-contractile tissue. This threshold was checked by visual interpretation and distribution of the greyscale values of the average of all greyscale graphs (tested with different cut-off values from 40–80, data not shown). This method is similar to the methods reported earlier in literature [11, 21]. The percentage of non-contractile tissue was calculated as the number of non-contractile pixels (grayscale value > 60) divided by the total number of pixels.

#### Demographics

Demographic variables including age, sex and body mass index (BMI) were obtained. For the statistical analysis, age and BMI were used as continuous variables.

#### Radiography

Weight-bearing, anterior–posterior radiographs of the knee joints were obtained in accordance with the Buckland-Wright protocol [32] and were used to determine radiographic severity of the knee OA in the index knee using the Kellgren-Lawrence grade (KL) [33]). Radiographs were scored, blinded, by an experienced radiologist.

### Statistical analysis

Descriptive analysis was used for participant characteristics including age, sex, BMI, radiographic severity, the percentage of non-contractile tissue, muscle strength and the GUG time. Normality of data was assessed using the Kolmogorov–Smirnov Test. Data sets of the primary outcome parameters muscle extensor strength and GUG time contained continues measures. The data of the percentage of non-contractile tissue were dichotomized due to skewness of the data into a low-percentage non-contractile tissue group and a high-percentage non-contractile tissue group, based on the median (11.2%), with equal group sizes (*n* = 47).

Subsequently, uni- and multivariable linear regression analyses were performed to determine the association of the independent variable percentage of non-contractile tissue with the dependent variables muscle strength and GUG time, controlled for the potential confounders (i.e., age, sex, BMI and radiographic severity (KL < 2 or ≥ 2)). Variables that changed the regression coefficient (B) of the percentage of non-contractile tissue by > 10% were considered to be confounders [34]. Interaction between the dichotomized percentage of non-contractile tissue and confounders was tested. All analyses were performed in IBM SPSS 22.0. A *P*-value of less than 0.05 was considered statistically significant.

## Results

Participant characteristics are shown in Table 1. A total of 94 participants were included in the analysis. The age and BMI (mean ± SD) were 61.7 ± 7.1 years and 29.1 ± 4.7 kg/m^2^, respectively. The sample was predominantly female (69%). Knee pain was scored as 5.2 ± 2.2 on the NRS. A KL grade of 2 or higher was found in 68% of the participants (i.e. radiographic knee OA).

**Table 1:** Participant characteristics for all included patients with knee osteoarthritis

	Mean ± SD
n	94
Age, years	61.7 ± 7.1
Sex (% female)	65 (69.1%)
BMI, kg/m^2^	29.1 ± 4.7
Radiographic severity (KL grade), n (%)	
0, 1	1 (1%), 29 (31%)
2, 3, 4	25 (27%), 21 (22%), 18 (19%)
Knee pain, NRS	5.2 ± 2.2
Duration complaints, years	11.4 ± 9.7
Percentage of non-contractile tissue (median, standard error, minimum—maximum, interquartile range)	11.2, 1.6, 0.1–63.0, 18.3
Vastus medialis thickness, mm	46.6 ± 7.5
Muscle strength (normalized to body weight), Nm/kg	0.9 ± 0.4
Get-Up and Go time, seconds	10.9 ± 2.3

*n* number of participants, *SD* standard deviation, *BMI* Body Mass Index, *NRS* numeric rating scale, *KL grade* Kellgren-Lawrence grade

The median of the percentage of non-contractile muscle tissue was 11.2% (interquartile range 18.3%, data right-skewed). Vastus medialis thickness was on average 46.6 ± 7.5 mm. Average muscle strength of the knee extensors (normalized to body weight) and GUG time were 0.9 ± 0.4 Nm/kg and 10.9 ± 2.3 s, respectively.

**Fig. 3:**
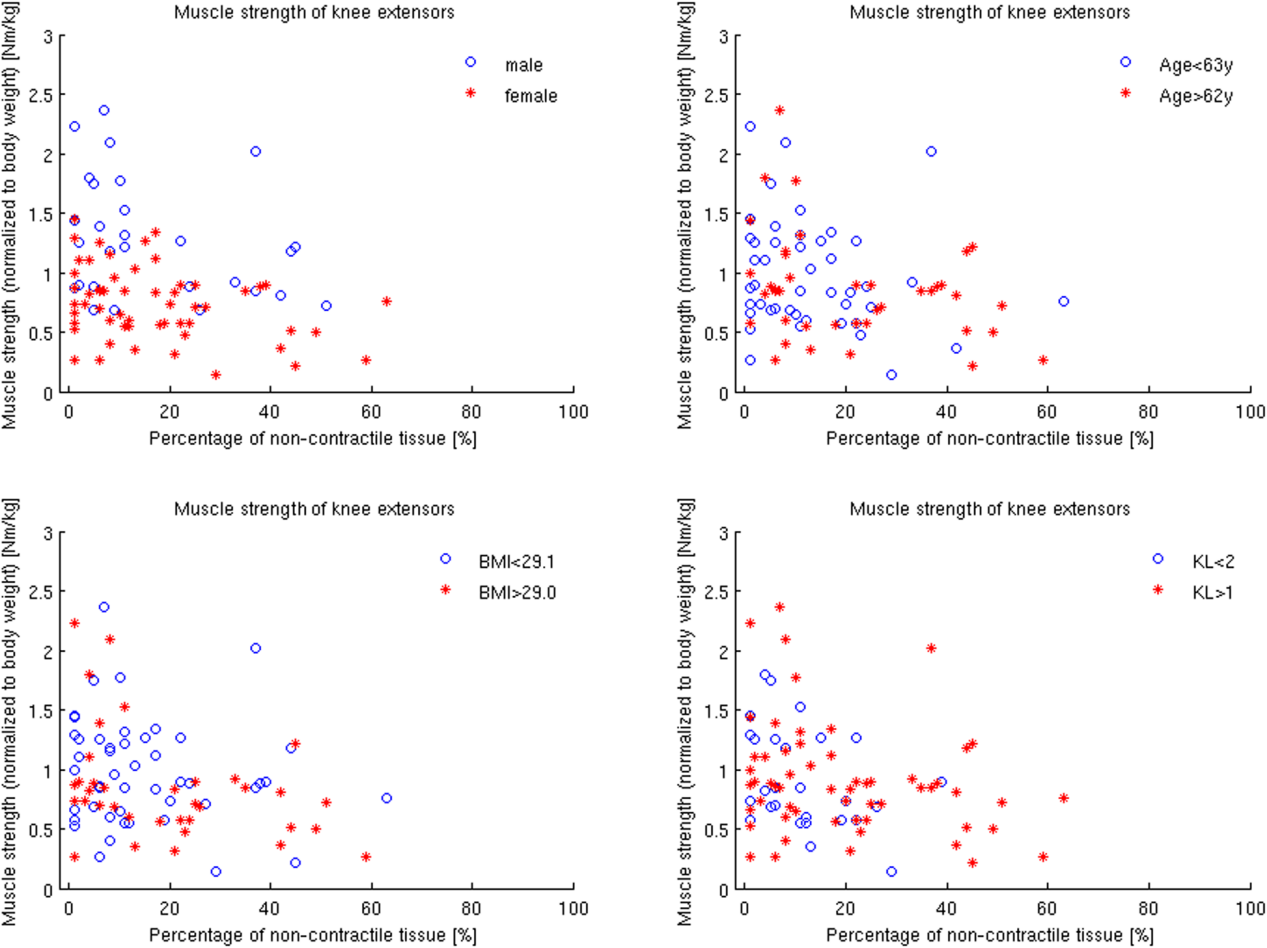
The individual data points of the percentage of non-contractile tissue of all patients with respect to the normalized muscle strength. The different symbols (o and *) represent the different confounders sex, age, BMI and radiographic severity

**Fig. 4:**
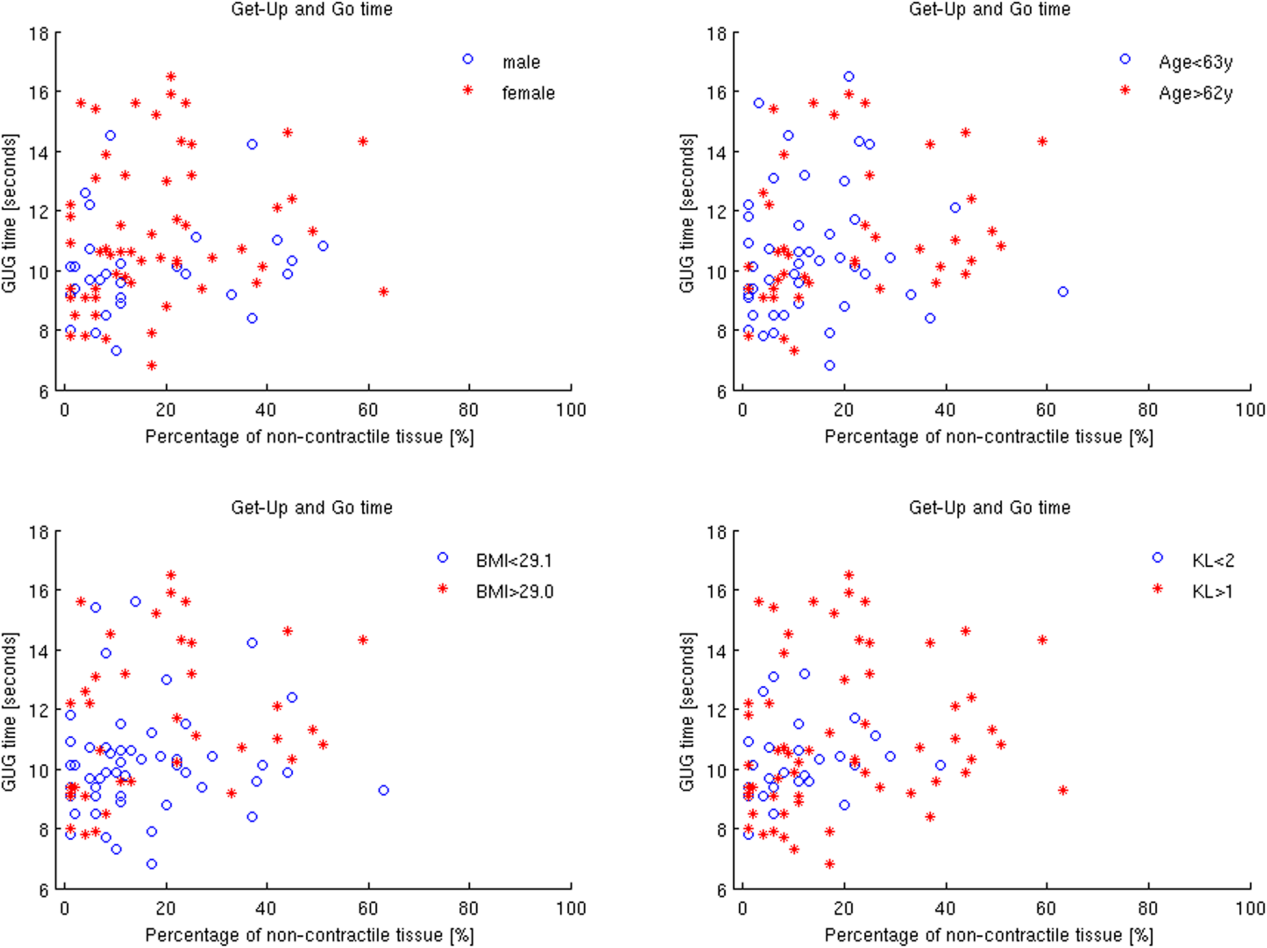
The individual data points of the percentage of non-contractile tissue of all patients with respect to the GUG time. The different symbols (o and *) represent the different confounders sex, age, BMI and radiographic severity

The percentage of non-contractile tissue of all patients with respect to the normalized muscle strength and to the GUG time is shown in Figs. 3 and 4.

Results from the regression analyses of the percentage of non-contractile muscle tissue on muscle extensor strength and GUG time are shown in Table 2. The percentage of non-contractile muscle tissue was found to be associated with normalized muscle strength (B = -0.25, *P* = 0.006) as well as with GUG time (B = 1.09, *P* = 0.021), implying a high percentage of non-contractile tissue (i.e. > 11.2%) is related to more pronounced muscle weakness and a longer GUG time.

**Table 2:** Uni- and multivariable linear regression analyses of the dichotomized percentage of non-contractile muscle tissue in the vastus medialis muscle on muscle strength of the knee extensors (normalized to body weight) and GUG time in patients with knee osteoarthritis. The multivariable analyses show the adjusted associations including the different possible confounders sex, age, BMI and radiographic severity

	Normalized muscle strength			GUG test		
	B	P	CI	B	P	CI
Unadjusted association, uni-variable analysis:						
Non-contractile tissue	-0.25	0.006^a^	-0.43 – -0.08	1.09	0.021^a^	0.17 – 2.01
Adjusted association, multivariable analysis including the four possible confounders: sex, age, BMI and radiographic severity:						
Non-contractile tissue	**-0.15**	0.049^a^	-0.30 – 0.00	**0.50**	0.248	-0.35 – 1.34
Sex	-0.52	< 0.001^a^	-0.67 – -0.36	1.09	0.017^a^	0.20 – 1.98
Age	-0.01	0.176	-0.02 – 0.00	0.07	0.022^a^	0.01 – 0.13
BMI	-0.02	0.005^a^	-0.04 – -0.01	0.20	< 0.001^a^	0.11 – 0.29
Radiographic severity	0.02	0.836	-0.14 – 0.17	0.41	0.364	-0.48 – 1.30
Adjusted association, multivariable analysis per confounder, to assess the effect on B:						
Non-contractile tissue	**-0.20**	0.009^a^	-0.35 – -0.05	**0.98**	0.035^a^	0.07 – 1.90
Sex	-0.51	< 0.001^a^	-0.67—-0.05	1.00	0.047^a^	0.01 – 1.99
Non-contractile tissue	-0.25	0.006^a^	-0.43 – -0.08	1.09	0.021^a^	0.17 – 2.00
Age	0.00	0.524	-0.02 – 0.01	0.05	0.115	-0.01 – 0.12
Non-contractile tissue	**-0.21**	0.025^a^	-0.39 – -0.03	**0.67**	0.130	-0.20 – 1.55
BMI	-0.02	0.031^a^	-0.04 – 0.00	0.19	< 0.001^a^	0.10 – 0.29
Non-contractile tissue	-0.26	0.006^a^	-0.44 – -0.08	1.03	0.029^a^	0.11 – 1.96
Radiographic severity	0.02	0.813	-0.17 – 0.21	0.67	0.183	-0.32 – 1.66

*B* unstandardized beta coefficient, *CI* 95% confidence interval, *GUG test* Get-Up and Go test, *BMI* body mass index

^a^*P* < 0.05, **bold** change of B-value of non-contractile tissue of > 10% due to confounder(s)

In multivariable regression analysis, the model with the four possible confounders showed a change in the unstandardized regression coefficient of the percentage of non-contractile tissue by > 10% in the association with muscle strength (B = -0.15, *P* = 0.049) and GUG (B = 0.50, *P* = 0.248). The association with muscle strength remained significant, but this did not apply to the association with GUG.

Sex changed the unstandardized regression coefficient of the percentage of non-contractile tissue with 20.0% in the association with muscle strength (B = -0.20, *P* = 0.009), and with 10.1% in the association with GUG time (B = 0.98, *P* = 0.035). Despite these changes, controlling for sex as a confounder still showed significant associations of the percentage of non-contractile tissue with muscle strength and GUG time (*P* = 0.009 and *P* = 0.035, respectively). The interactions between the percentage of non-contractile tissue and sex for muscle strength and GUG time were not significant (*P* = 0.362 and *P* = 0.511, respectively). Average muscle strength and GUG time for females were 0.7 ± 0.3 Nm/kg and 11.2 ± 2.5 s, and for males 1.3 ± 0.5 Nm/kg and 10.1 ± 1.7 s, respectively.

BMI changed the unstandardized regression coefficient of the percentage of non-contractile tissue with 16.0% in the association with muscle strength (B = -0.21, *P* = 0.025), and with 38.5% in the association with GUG time (B = 0.67, *P* = 0.130). The interactions between the percentage of non-contractile tissue and BMI for muscle strength and GUG time were not significant (*P* = 0.605 and *P* = 0.386, respectively).

Age confounded the association with GUG (*P* = 0.022) but not the association with muscle strength (*P* = 0.176). However, age did not change the unstandardized regression coefficient of the percentage of non-contractile tissue in the association with GUG time (B = 1.09).

Radiographic severity did not contribute to the confounding of either associations.

## Discussion

Patients with a high percentage of non-contractile muscle tissue of the vastus medialis (i.e. > 11.2%), showed a more pronounced extensor muscle weakness (0.25 Nm/kg) and a longer GUG time (1.09 s). The associations were confounded by the variables sex and BMI. Age also contributed in the association with GUG. Due to the confounders the association with GUG did not remain significant. Radiographic severity of OA did not contributed to the confounding of either of the associations. Measurement of muscle tissue composition with clinical routine MRI can be considered as an important contribution to a better understanding of the underlying mechanisms of muscle weakness in knee OA.

Conflicting results have been reported on the association between non-contractile muscle tissue and muscle weakness [14–19]. In healthy males (*n* = 9), non-contractile muscle tissue (measured using a chemical shift-based water/fat separation MRI method) was shown to correlate with weakness of the quadriceps [15]. Further, significant correlations were observed in healthy subjects (*n* = 20) between fat fraction and relative maximum voluntary contraction of the quadriceps (also using a chemical shift-based water/fat MRI method) [16]. Also in elderly (*n* = 2627), greater fat infiltration (measured using computer tomography) of the thigh muscles was associated with muscle weakness [17], and with poorer physical function (*n* = 2979) and increased risk of mobility loss (*n* = 2631) [18, 19]. In knee OA, Kumar et al. (*n* = 96) found that quadriceps intramuscular fat fraction (measured using a chemical shift-based water/fat separation MRI method) was not associated with quadriceps weakness [14]. However, they found associations with physical function, showing a shorter distance at the six minute walk test and a longer time on the stair climb test in the presence of a high intramuscular fat fraction in the quadriceps [14]. Interestingly, in knee OA Wada et al. (*n* = 906) found an inverted U-shaped relationship between body fat mass (measured using bio-impedance) and quadriceps strength [35]. Independent of fat mass, leg muscle mass (also measured using bio-impedance) was linearly associated with greater quadriceps strength. They concluded that a separate assessment of fat mass and muscle mass is required in patients with knee OA.

Our results on the GUG test as a functional measurement of knee muscle strength are comparable with the findings of Kumar et al. [14] on physical function. However, our outcomes on muscle extensor strength are not comparable to their findings on quadriceps weakness. The differences in results can be explained by differences in study population and assessment methods. In Kumar et al., a number of participants were diagnosed with a KL grade of ≤ 1 and identified as controls, in contrast to our study in which each KL grade 1–4 included a comparable number of subjects. Also, they used a chemical shift-based water/fat separation MRI method and assessed the whole quadriceps muscle group, segmenting a 2 cm section between 10-12 cm proximal to the superior pole of the patella. We focused on clinically available T1-weighted MRI of the vastus medialis muscle only, using a standardized region of interest (ROI) for analysis. The vastus medialis as part of the quadriceps supports knee extension and stabilizes the patellofemoral joint, which is only part of the total function of extension by the quadriceps. Furthermore, Kumar et al. measured isometric torque at 70° knee flexion, and maximal isokinetic torque at 120°/sec between 20–90° [14], whereas we measured isokinetically at 60 ^o^/sec between 20–80° of knee extension.

The associations of the percentage of non-contractile tissue with muscle extensor strength and GUG time were specifically confounded by sex and BMI. It is known that women have a lower muscle strength and are more at risk for OA [2, 36], which might explain these findings. The role of high BMI has been addressed in other studies (e.g. [35, 37]), suggesting that a high BMI is associated with a high percentage of fatty infiltration in the muscle, particularly when patients avoid daily activities [20]. This indicates that women with a high BMI are more at risk for having a high amount of non-contractile tissue in the muscle.

Radiographic severity and age were not found to be confounders in the determined association with muscle strength. The observation that radiographic severity did not contribute to the confounding of either association is important and suggests that muscle strength is not associated with radiographic severity, which is in line with previous findings [38, 39]. Age was only a confounder in the association with GUG, but did not change the regression coefficient with > 10%. Kumar et al. [14] did adjust for age, sex, BMI, OA severity and found that a higher quadriceps intramuscular fat fraction was associated with greater severity of OA and with older age. As mentioned before, their study population and assessment methods were somewhat different. The fact that their control group (KL ≤ 1) was younger than the radiographic OA group might have influenced the results. Furthermore, they reported a small difference of 1.5% in fat infiltration between groups of OA severity, which might not have been visible on the sagittal T1-weigthed MRI we used.

Previously, muscle strength has been associated with muscle mass and cross sectional area as measures of muscle quantity [9–11]. Assessment of non-contractile muscle tissue may contribute to a better understanding of the mechanisms underlying muscle weakness. The use of information from MRI in relation to muscle weakness in knee OA may be very relevant to the clinic. Next to the assessment of joint tissues in knee OA (joint space, cartilage, meniscus, bone marrow lesions, ligaments, synovium), assessment of muscle composition might add information to research and clinical practice.

In this study, clinically available sagittal T1-weigthed 3T MRI was used for the assessment of the percentage of non-contractile tissue in the vastus medialis. This MRI method provides the possibility to analyze the composition of muscle tissue visually [20] or by using simple quantification methods [11, 21]. However, it is not possible to further separate the various components of non-contractile tissue, in contrast to the more advanced water/fat separation MRI methods.

The ROI used to determine the grayscale values for muscle tissue analyses, consisted of a standardized area dependent on the muscle thickness. The field of view of the clinical MRI images was not large enough to bring the entire vastus medialis in image and therefore a precise determination of the thickness of the muscle, of the cross-sectional area, or a segmentation of the whole muscle, was not possible. This indicates that information with regard to the total amount or (possible inhomogeneous) distribution of non-contractile muscle tissue was unknown. Other studies also used ROIs of a part of the muscle [12–14, 21]. The benefit of using the ROI is that the assessment was standardized for all patients, and shown to be reliable between raters. A limitation of using the ROI in this way might be that the location is dependent of the length of the subject, with longer subjects having the ROI placed more distally with respect to the whole muscle length. For future studies, the use of a larger ROI, multiple ROIs, or segmentation of the whole muscle is recommended to draw conclusions regarding the distribution and the total amount of non-contractile muscle tissue in the muscle.

A limitation of this study might be that the grayscale threshold, used to distinguish between contractile and non-contractile muscle tissue in the images, was set arbitrarily. To overcome this, the threshold was checked by visual interpretation and distribution. For future studies it would be of interest to explore the method described by Kent-Braun et al. (21) in which the threshold was determined in a ROI consisting of about 50% subcutaneous fat and 50% muscle tissue and applied to a different RIO. Further, the choice for the cut-off point for dichotomization of the percentage of non-contractile muscle tissue of 11.2% was an arbitrary choice based on the median of the data. It can be debated whether this truly represents low- versus high-percentage non-contractile tissue groups. Also, in the analyses we normalized muscle strength to body weight (Nm/kg), as has been done more often in studies. Normalization of muscle strength to cross-sectional area of the muscle might better reflect the muscle mass. However, the cross-sectional area of the muscle belly was not available in these clinical MRI’s as mentioned before.

This paper focused on the vastus medialis muscle and measured muscle strength by knee extension on the dynamometer. Assessment of other muscles relevant for knee OA (e.g. vastus lateralis and hamstrings) and their related movements (e.g. knee flexion) would provide a more complete overview of the general relationship between the percentage of non-contractile muscle tissue and muscle weakness in knee OA and should therefore be subject of future studies. Also other possible confounders, such as the activity level of the patients, would be of interest to take into account in future studies.

## Conclusions

High-percentage non-contractile tissue in the vastus medialis muscle measured by clinical T1-weighted 3T MRI is associated with more pronounced muscle weakness, but the association is specifically confounded by sex and BMI. A high percentage of non-contractile muscle tissue seems to be an important compositional property of the vastus medialis muscle underlying quadriceps weakness.

## Data Availability

The datasets used and/or analysed during the current study are available from the corresponding author on reasonable request.

## Abbreviations

OA: Osteoarthritis
MRI: Magnetic resonance imaging
GUG: Get-Up and Go test
NRS: Numeric rating scale
ROI: Region of interest
B: Regression coefficient
BMI: Body mass index
